# Advances in the Measurement and Interpretation of Intervertebral Motion in the Lumbar Spine: A Scoping Review

**DOI:** 10.3390/bioengineering13020239

**Published:** 2026-02-18

**Authors:** Alan Breen, Alexander Breen, Jonathan Branney, Alister du Rose, Mehdi Nematimoez

**Affiliations:** 1 Faculty of Science and Technology, Bournemouth University, Poole BH12 5B, UK; alexbreen@bournemouth.ac.uk; 2 Faculty of Health, Environment and Medical Sciences, Bournemouth University, Poole BH8 8GP, UK; jbranney@bournemouth.ac.uk; 3 AECC School of Chiropractic, Health Sciences University, 13–15 Parkwood Road, Bournemouth BH5 2DF, UK; adurose@aecc.ac.uk; 4 Institute for Advanced Biomechanics and Motion Studies, Offenburg University, Max Planck Str. 1, 77656 Offenburg, Germany; nemati80@gmail.com

**Keywords:** intervertebral motion, measurement, interpretation, lumbar spine, human

## Abstract

Background: Intervertebral motion is a fundamental aspect of spinal biomechanics, crucial for understanding lumbar spine function, pain mechanisms, and surgical outcomes. Various methods exist for measuring and interpreting it, each with its own advantages, limitations, and specific applications. However, a comprehensive and standard taxonomy of study types for the measurement and interpretation of in vivo intervertebral motion in the lumbar spine is lacking. Objectives: This review aimed to systematically identify, characterise, and categorise the diverse study types deposited in the literature. Eligibility criteria: Only studies in English and of lumbar spine intervertebral motion in living subjects were considered, and only those that employed objective measurement of motion sequences were included. Sources of evidence: A comprehensive literature search was performed in PubMed, CINAHL, and SCOPUS for articles published between January 2000 and October 2025. Charting methods: After removal of duplicates, all studies were subjected to Title and abstract screening, followed by full-text screening of potentially eligible studies. Data selected were charted into tables under the headings: author, year, country, purpose, technology, participants, measurement, interpretation, radiation dosage, and significance of findings. Results: Forty-nine studies were abstracted and are described under 11 study types. These formed a taxonomy constituting the following six categories: normal biomechanical mechanisms, pathological and injury mechanisms, direct kinematic measurement, spinal stabilisation, dynamic radiography, and clinical markers. The resulting taxonomy will serve as a resource for researchers, clinicians, and policymakers by facilitating a more coherent understanding of the field and promoting standardisation in research design and reporting.

## 1. Introduction

**Figure 1:**
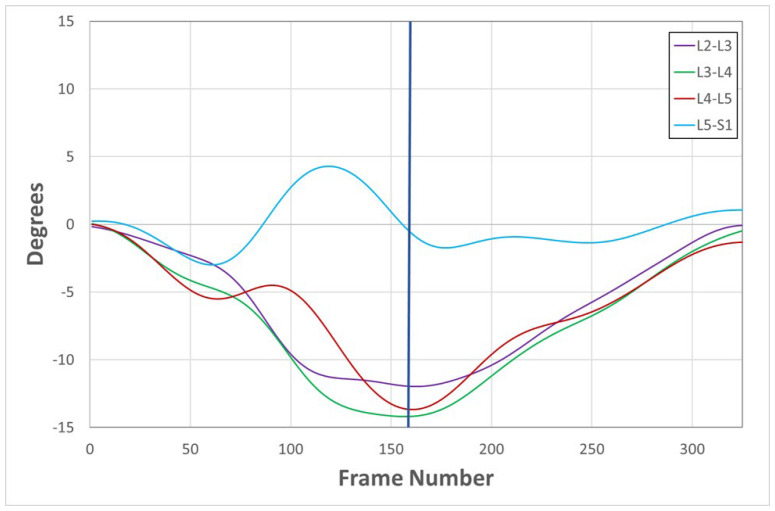
Intervertebral rotation graph of L2-3 to L5-S1 motion during standing flexion and return in an asymptomatic control participant measured using quantitative fluoroscopy (QF). (Blue vertical line indicates point of maximum global flexion.) L5–S1 exhibits anti-directional motion in its outward path, returning to its starting position by the end of its global range. (Source: Breen, A.; Breen, A.; Reference Database of Continuous Vertebral Flexion and Return. Open Science Framework 2022, https://doi.org/10.17605/osf.io/a27py). A video of the motion sequence depicted in this graph can be found in the Supplementary Materials.

The scientific literature pertaining to low back pain is large and heterogeneous. It contains many studies relating to intervertebral motion, yet these sometimes seem to lack coherence. Researchers who study intervertebral motion have increasingly noticed discrepancies in the design and reporting of studies resulting from an inadequate definition of the phenomenon. This may ignore the temporal component [1], and/or ignore the path that the motion has taken [2], and/or use only the global start and stop positions to calculate the range of motion [3]. As an illustration, Figure 1 shows intervertebral rotation from a healthy asymptomatic research participant in which the L5 vertebra has returned to its starting position by the end of the participant’s maximum bending range, falsely indicating “no motion” at that level. The example also demonstrates the extent to which spatiotemporal interactions within a group of motion segments may appear conflicting but are not associated with any pain or disability.

Methods for measuring and interpreting such motion have reached higher prominence in the research literature as technological sophistication has progressed, and some expect that we are on the verge of making important advances [4,5,6]. However, clinical studies still tend to confine intervertebral motion to overall rotation in upright postures, ignoring, for example, incremental rotation, translation, velocity, and the sequencing of motion onsets. Passive restraint is seldom mentioned in studies of in vivo lumbar motion, but it is a necessary option in the investigation of the types of instability that could be obscured by loading and muscle activity.

Authors also sometimes imply that landmarks on the surface of the back over the lumbar spine represent the lumbar spine itself or compare (or combine) intervertebral motion findings with studies that have used different data collection protocols [7,8]. These discrepancies introduce uncontrolled variation (noise) that can obscure comparisons [9]. Thus, a comprehensive and standardised taxonomy of study types for the measurement and interpretation of in vivo intervertebral motion in the lumbar spine is needed if we are to compare findings across studies, synthesise evidence, and guide future research.

In particular, we wish to point out at the outset of this review that motion is the process of an object changing position over time, while displacement specifically refers to the change in position from an initial point to a final point, including direction. Thus, motion can be described using concepts like velocity and acceleration. By contrast, displacement is the change from an initial to a final position (spatial only) and the distance and direction between these positions. It follows that intervertebral range of motion is the maximum displacement between a pair of vertebral segments during a specific motion in a given plane and not, as the long-held definition states, “the difference between the two points of physiologic extent of motion” [10]. Instead, we contend that it can occur anywhere in the motion and not necessarily at the end of the path of any given section of the spine that contains the vertebral pair. Thus, “motion” is defined as “change in location from a spatial position A to a different position B, whereby the moving figure was located at position A at time T1 and then located at position B at another time T2” [11]. Talmy (1984) considers the path of motion to be the fundamental component of a motion event because, without a path, there is no motion [11]. In the case of motion events, a change in spatiotemporal location means a change in the spatial and temporal configuration of the path that is the result of the motion [12].

This scoping review aimed to address these issues by systematically identifying the study types employed in the literature to measure and interpret intervertebral motion. The intention was to map the prevailing concepts and create a taxonomy of studies that have involved the measurement and interpretation of in vivo lumbar spine intervertebral motion over the past 25 years. This information will hopefully serve as a resource for clinicians, researchers, and policymakers to help facilitate a more coherent understanding of practice in the field and promote high standards in research design and reporting [13].

## 2. Materials and Methods

This scoping review was conducted in accordance with the Arksey and O’Malley (2005) framework and following the PRISMA-ScR (Preferred Reporting Items for Systematic Review and Meta-Analyses (extension for Scoping Reviews) guidelines [14,15]. A protocol was preregistered on the OSF database (https://osf.io/dnwua/overview, Center for Open Science, Charlottsville, VA, USA) and a literature search was conducted by one author using the electronic databases: PubMed, SCOPUS and CINAHL Ultimate, with Boolean operators combining MeSH terms and key words relating to: “lumbar spine”, “intervertebral motion”, “measurement”, “interpretation”, “human” and “in vivo”. Using the bibliographic search software: (https://rayyan.ai/, v5.4.0, Rayyan Systems Inc., Cambridge, MA, USA) reports were identified and imported into a ReadCube reference management database (https://papersapp.com, Digital Science & Research Solutions Ltd., Cambridge, MA, USA) for screening.

Eligible studies were published between January 2000 and October 2025 and involved human participants of any age group, where in vivo intervertebral motion of the lumbar spine (L1–S1) was measured and interpreted. Systematic reviews during this period were accessed and screened for additional eligible studies. Studies of interest generally encompass kinematics (e.g., range of motion (RoM), instantaneous centre of rotation (ICR), coupled motions), dynamics (e.g., motion patterns under load), and the methodologies used to acquire, analyse, and/or interpret these data. The context was any clinical or research setting in which in vivo lumbar intervertebral motion was studied. This included, but was not limited to, studies of healthy individuals, patients with low back pain, post-surgical patients, athletes, and people in occupational settings. An example search string for PubMed might include:

“(lumbar spine[MeSH Terms] OR lumbar vertebra* OR lumbosacral OR low back) AND (intervertebral disc[MeSH Terms] OR intervertebral motion OR spinal motion OR kinematics OR dynamics OR range of motion OR coupled motion) AND (in vivo OR living OR human) AND (measurement OR imaging OR fluoroscopy OR MRI OR CT OR X-ray OR radiography OR optical tracking OR motion capture OR video analysis OR wearable sensor*)”

All empirical study designs were considered (e.g., observational studies, experimental studies, case series, cohort studies, cross-sectional studies). Narrative reviews, theoretical papers, animal studies, in vitro studies, cadaveric studies, and biomechanical models without in vivo validation were excluded. Studies were eligible if they satisfied all the following: Quantitative measurement, Continuous intervertebral motion (>3 Hz), Lumbar spine, In vivo, Human, and Article in English. Studies considered ineligible were: Reviews, Book chapters, Commentaries, Case studies, Qualitative studies, Discussions, Editorials, Letters, Guidelines, Protocols, and Conference papers.

After removal of duplicates, all studies were subjected to title and abstract screening for eligibility by the lead author and one other, and a subset was identified for full-text screening. Cases of disagreement were arbitrated by a third author. This procedure was also followed for the charting process, in which information from studies eligible for full-text screening was initially extracted into a spreadsheet under the headings: lead author, year, short title, qualifying descriptions, include/exclude (with reasons), and access information (e.g., DOI). The results were considered by a different author and, in the event of disagreement, arbitrated by a further one before being allocated to their final charting position. Any additional articles chosen from private databases that did not appear in the search were considered in the same way at this stage.

Data items from studies that were accepted for inclusion were charted into tables according to their type and under the descriptive headings: author/year, country of lead author, purpose of study, technology, participants/gender, measurement, interpretation, radiation dose (if applicable), and significance of findings (see Supplementary Materials). Studies were not assessed for methodological quality beyond requiring correct nomenclature. However, studies were only charted if they represented peer-reviewed and published research that met the criteria for eligibility and inclusion as independently verified by three authors. The charting process strove to present information from the studies as distinctive types rather than to evaluate them. All authors approved the results of the charting process.

## 3. Results

**Figure 2:**
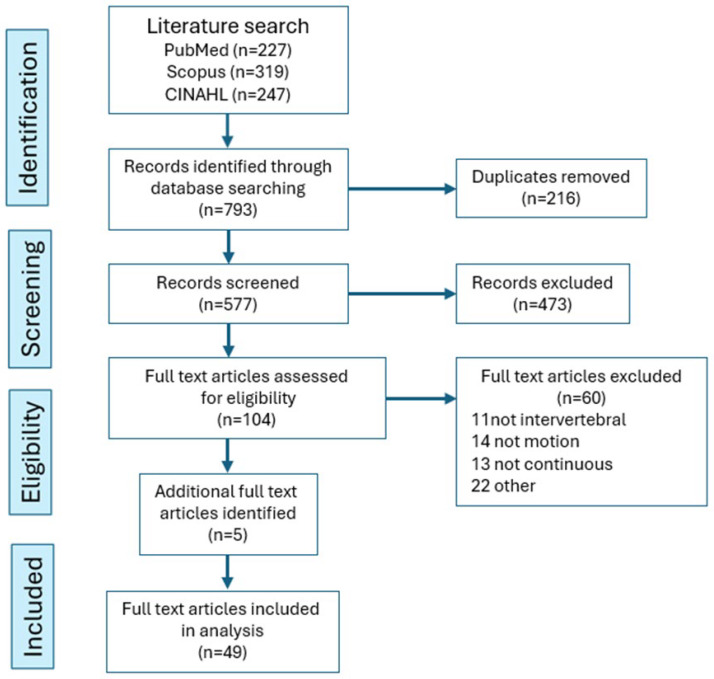
PRISMA flowchart.

**Figure 3:**
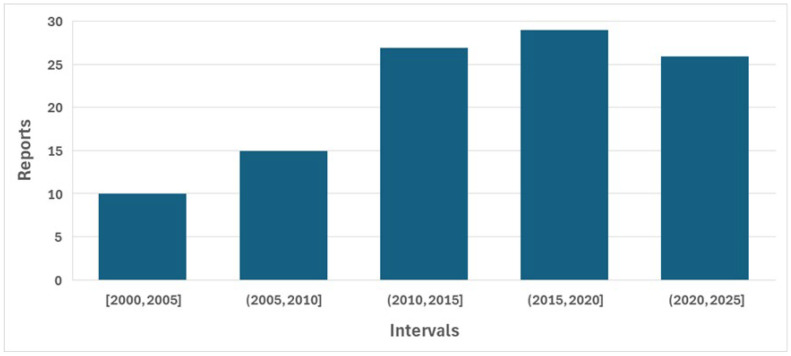
5-year distribution of publication dates (2000–2025) of the 104 studies that underwent full-text screening.

Database searching identified 793 studies, of which 216 were duplicates and were removed, leaving 577 for consideration (Figure 2). After title and abstract screening, 473 of these were excluded, leaving 104 for full-text screening. Of these, 60 were excluded, mainly because they did not study intervertebral motion (11), did not study motion (14), or did not study continuous motion (13). After the addition of five studies from other sources known to the authors, and following full-text screening, 49 studies were found to be eligible for review. The 5-year distribution of publication dates of the 104 that underwent full-text screening shows an approximate doubling of articles that were published 5 years after the first decade of the study period (Figure 3).

The 49 studies that met the criteria for eligibility and inclusion are represented as 11 types and shown in Table 1. These had a total of 2151 participants with lead authors from 11 countries: USA (17), UK (15), Japan (3), China (3), Canada (3), Switzerland (2), Italy (2), Australia (1), Finland (1), Germany (1), and Iran (1).

### 3.1. Intervertebral Motion Interactions

Among the first phenomena to be studied in relation to the interactions between the intervertebral motion segments (and that occurred either prior to the review period, and not therefore part of the results [16], or during it [17]), were the relative onsets of angular intervertebral motion during bending. Like many of the studies in this review, motion X-rays were used to register the kinematics, which initially presented some technological barriers to measurement that had to be overcome to progress the work. It was found that after the initial Japanese studies, some 20 years had elapsed before automated vertebral image tracking and digital imaging improved the analysis process sufficiently to encourage phase lag studies to recommence. When it did, the value of measuring motion contributions for making comparisons between individuals or groups, rather than raw values that were prone to wide variation, became apparent [18]. Then, to further explore apparent kinematic biomarkers for chronic, nonspecific back pain, principal component analysis was used [19]. This was followed by the classification of the motion path types originally discovered by Harada et al. [17], using cineradiography, but now incorporating the first derivatives (velocity) of sagittal plane intervertebral rotation [20]. This demonstrated, through cluster analysis, that the top-down cascade pattern of lumbar flexion referred to by Harada in 2000 [17] appeared to be the most common pattern of “phase lag”. However, clinical studies are needed to understand the relevance of this in symptomatic states.

### 3.2. Disc Degeneration Kinematics

The chronology of the use of intervertebral motion in the study of disc degeneration began with Takayagani et al., 2001 [21], comparing flexion and return for rotation and translation in 41 patients with L4 degenerative spondylolisthesis and 20 controls. To the best of our knowledge, this was the first study to report on spondylolisthesis kinematics. It found that rotation and translation were simultaneous in controls, but not in patients, where the more severe cases exhibited decreased translation. Following this, 7 years later, Hasegawa et al. [22] used an intraoperative spinous distraction apparatus to measure restraint in terms of absorption energy and the neutral zone (NZ), taking disc height loss into account. The authors found that “Stiffness demonstrated a significant negative and NZ a significant positive relationship with disc height”. However, there were no significant differences between spines with “collapsed” discs than those without, although the NZ value was higher in those without the collapsed types and more sensitive to this measure in degenerative segments with preserved disc height. The authors went on to suggest that “degenerative segments with preserved disc height have a latent instability compared to segments with collapsed discs”. These two studies illustrate an interesting comparison of the kinematic versus the loading effects of disc degeneration.

Following the appearance of quantitative fluoroscopic systems, Breen et al. [23,24] compared the kinematics of non-spondylolisthesis patients who had early-to-moderate disc degeneration to pain-free controls in both standing and lying flexion-extension. The authors’ intention was to test the hypothesis of Farfan and Kirkaldy-Willis that early disc degeneration is associated with instability [25]. This study found that patients with disc degeneration exhibited more disrupted interactions between segments in terms of motion sharing, but only in passive recumbent motion. There was also only a weak-to-moderate negative correlation between disc-height loss and weight-bearing RoM, and no correlation with translation or laxity. Meanwhile, Dombrowski et al. [2], using standing 3D fluoroscopy and CT-generated bone models to study continuous dynamic sagittal rotation in degenerative spondylolisthesis, compared their findings with those from static flexion-extension radiographs. Their study found that 42% of the spondylolisthesis group demonstrated aberrant mid-range motion on intervertebral dynamic motion analysis and greater flexion translation on dynamic than static imaging. Caution is advised, however, in the interpretation of these latest studies, none of which assessed more than 10 patient-control pairs each.

### 3.3. Implanted Markers and Internal Sensors

Implanted marker systems in the spine are generally considered too invasive for other than serious spinal disorders, however they are still in use, although more usually for the measurement of displacement using static radiographs than for the measurement of actual intervertebral motion in disc replacement surgery [26]. However, to capture motion and avoid radiation, implanted wires and screws have also been used to mount sensors percutaneously for tracking by motion capture systems.

Five studies were found in this category, two of chronic back pain patients [27,28] and three of healthy controls [29,30,31]. All used optical sensors were attached percutaneously to pedicle screws or K-wires implanted into the spines of chronic back pain patients, and no further ones seem to have been published since 2013. The patient studies used 3D and 2D analysis, respectively, and did not involve control groups. Their authors suggest that they could be useful in testing internal fixation systems where the spine is being surgically exposed.

One system [29] used ultrasound tracking of LEDs from L12–L2 and found within-subject variation of 0-6-42% during flexion-extension, lateral bending, and axial rotation, respectively. The last two studied normal ranges of the same motions from L1–S1 (the first study to compare the whole lumbar spine in vivo using bone pins) [30] and reported that the main intervertebral motion during gait is mid-lumbar and coronal [31].

### 3.4. Assessment of Lumbar Orthoses

Two early US studies [32,33] were found to have used 2D quantitative fluoroscopy in small groups of controls (4 and 10, respectively). Both used free (uncontrolled) bending during flexion/extension. The first tested a custom-fitted thoracolumbosacral orthosis and found that it reduced flexion range by 2/3, while the second compared no orthotic, a soft orthotic, a semirigid orthotic, and a semirigid thoracolumbosacral orthotic and found that all three devices limited L3–4 and L4–5 flexion, but not L5–S1.

### 3.5. Technology Development

The 10 studies identified mainly challenged the technical viability of the technologies in this review for reliability, accuracy, and radiation dosage during controlled versus uncontrolled weight bearing and recumbent flexion, extension, and lateral bending. These mainly employed 2D quantitative fluoroscopy [7,34,35,36,37,38,39,40,41] with one study of the reliability of an implanted Kirshner wire system for measurement of 3D L1–S1 motion during gait [42]. This study reported similar findings to Mac Williams et al. [31] (see above). Studies were mainly conducted by US and UK groups, also with representation from Italy and Canada.

### 3.6. Back Pain Biomarkers

In their review of biomarker types in relation to chronic pain, Reckziegel et al. [43] concluded that the field has been advancing in terms of the investigation of hypotheses pertaining to underlying mechanisms. For chronic, nonspecific low back pain, the tendency has often been to frame these hypotheses around disordered intervertebral motion. The chief suspects have been excessive angular and/or linear RoM, laxity (as reflected by a steep starting motion gradient or attainment rate), or an increased attainment rate around the mid-range of the intervertebral motion path—suggestive of instability.

In our review, we found no clear evidence for excessive RoM, whether using static or dynamic radiographs [44]. However, the attainment rate during standing flexion was assessed by Teyhen et al. [45] using 2D quantitative fluoroscopy, who found it to be increased, although still with no evidence of increased RoM. A subsequent subgroup study comparing chronic nonspecific back pain patients and controls in flexion and return [46] found a significantly smaller rotational motion contribution during the return phase from standing flexion at L5–S1 in patients.

Similar 2D fluoroscopy studies using passive recumbent flexion did not detect this [23,47,48] but instead found greater inequality of motion sharing (MSI) in patients with chronic nonspecific back pain. A further passive recumbent flexion and return study probed this further by measuring the timing of peak velocity during intervertebral flexion. This was found to occur earlier in the motion path at L5–S1 in back pain patients than in controls [49]. Taken together, these studies may indicate MSI as a biomarker, linking disruption of passive restraint and chronic back pain.

Meanwhile, a recent 3D quantitative fluoroscopy study with CT models that assessed standing flexion-extension and lateral bending in patients with chronic back pain measured intervertebral rotations, translations, and coupling and found heterogeneity in these variables [50]. This study did not have a control group, and further research is required. Another recent biplanar fluoroscopy study using 3D CT models examined the movement of endplate centres with respect to the sacrum before and after lifting in patients with recurrent back pain and healthy controls [51]. This study found significant inter-test differences, with and without fatigue, in translation and *z*-axis rotation in both groups. This occurred slightly later in flexion-extension motion after fatigue. The authors suggest that this may indicate a protective mechanism or a role in dysfunction.

### 3.7. Post Stabilisation Dynamics

Only two studies were found that addressed this. The first presented preliminary data and used biplanar fluoroscopy to track implanted metal markers in five patients, 2, 3, and 6 months post-fusion, comparing intervertebral displacement at the beginning and end of trunk motion with minimum/maximum intervertebral displacement over the motion path, however, these did not correspond [3].

The other study [52] used 2D fluoroscopy in 24 patients with flexible total disc replacements (TDRs) 6 weeks and 5 years post-surgery. The upper and lower TDR endplates were tracked, and the continuous RoM with 10 repeated cycles was measured in the coronal and sagittal planes during treadmill walking, indicating partial preservation of motion.

### 3.8. Normative Lumbar Kinematics

**Figure 4:**
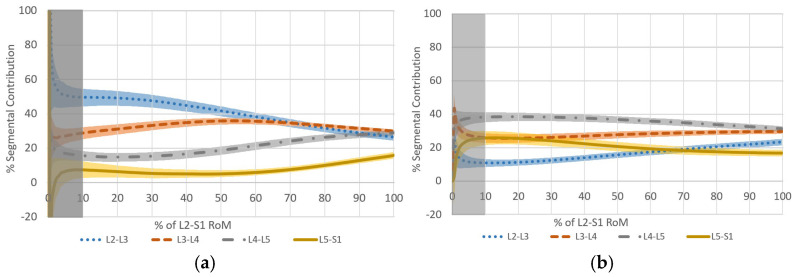
Average segmental contributions (L2–3 to L5–S1) during (**a**) standing flexion and (**b**) return in 127 healthy controls. Shaded areas indicate 95% confidence intervals. (from: Breen, A.; Breen, A. Reference Database of Continuous Vertebral Flexion and Return. Open Science Framework 2022, https://doi.org/10.17605/osf.io/a27py). (Grey shading over the first 10% of the motion in both graphs indicates high error areas to be ignored due to small values in the percentage calculations).

Five studies were eligible for the reporting of in vivo normative intervertebral motion. All were from the authors’ own labs, and all used the same 2D fluoroscopy technology protocols [53]. They cannot, therefore, be extrapolated to other systems. A normative database is presented, consisting of 127 anonymized pain-free controls imaged during controlled weight bearing with recumbent left, right, flexion, and extension lumbar motion from vertebral midplane angles throughout the motion sequences as captured at 15 fps. These data are publicly available from the Open Science Framework database (https://osf.io/a27py/) [46]. They can be used to display and manipulate the motion sequence data with the appropriate transformations for comparison with users’ own studies, if desired. For weight-bearing flexion and return, transformation of intervertebral angles to proportional contributions has revealed the level-by-level normative motion contribution patterns as a phenotype for comparison with clinical and experimental studies [46,54] (Figure 4).

The other studies present the lumbar L2–S1 IV-RoM, translation, laxity, MSI, MSV, regularity, and symmetry from individual studies of healthy control populations in different configurations. All studies present their effective radiation dosage. Two studies present the minimal detectable change and intrasubject repeatability in healthy controls of some of the studies over a 6-week period [55,56]. These show evidence that measurements of translation and MSV are not suitable for longitudinal studies and that surface EMG measurements of longissimus thoracis, lumborum, and multifidus amplitudes correlate with maximum RoM at L4–5 and L5–S1 during controlled standing flexion and return motion but are essentially silent during passive recumbent flexion and return.

### 3.9. Intervertebral Force-Deformation

Four studies were found. All were of healthy controls and involved bending and lifting functional loads. All used 3D fluoroscopy linked to CT-generated bone models, one measuring sagittal intervertebral rotation and translation [57] and another measuring average ICR locations and migration ranges [58]. These two found linear relationships between load, rotation, translation, and level-specific changes in ICR location and dispersion with increased loading. In the future, this information could contribute to the establishment of biomechanical models and normative values with which to compare conditions and interventions [59].

One of the other studies investigated changes in disc height and shear strain patterns and distributions during lifting [60], finding that these restraint patterns can be mapped and could contribute to models. The last study tested facet joint kinematics during lifting and found greater translation at L3 and L4 than at other levels in response to loads, which may help to explain the pathogenesis of structural changes associated with load bearing [61].

### 3.10. Aerospace Lumbar Kinematics

Two studies addressed the problem of post-spaceflight disc herniation in relation to lumbar spine kinematics using 2D quantitative fluoroscopy and MRI. The first examined relationships between prolonged exposure to microgravity and weight-bearing flexion kinematics in 12 ISS crew members, six of whom developed disc hernia symptoms post-flight [62]. This study found, with MRI, that post-spaceflight disc herniation was associated with compromised multifidus quality and, using 2D fluoroscopy, that symptomatic returning astronauts had reduced flexion-extension RoM at their L3–4 and L4–5 levels.

The second study tested a microgravity countermeasure skinsuit that compressed the spine overnight to limit the intervertebral stiffness that follows a period of absence of axial loading and preserves motion in the spine. Twenty healthy controls were tested with and without skinsuit use, finding significantly more lumbar intervertebral flexion and translation range after the skinsuit was used [63]. These studies may have implications for longer space missions, including interplanetary travel.

### 3.11. Soft Tissue Artefact (STA) Measurement

Three recent studies attempting to measure soft tissue artefacts were eligible for this review. The first [64] compared optical motion capture in six controls for flexion and extension, with one landmark’s motion measured with 3D quantitative fluoroscopy driven by CT models. With this protocol, STAs for flexion ranged from 4.0 mm for L1–3 to 13.5 mm for L4–5, and for extension, 2.7 mm for L4–5 and 6.1 mm for L1–3. Errors varied with anatomical direction, marker location, vertebral level, and bending phase.

The second study [8] aimed to evaluate static placement errors as well as STA from MoCap optical marker clusters during flexion-extension and lateral bending in 39 low back pain patients, taking patient characteristics into account. Data from surface markers positioned over L1 and L5 spinous processes were compared to continuous data from 3D fluoroscopy with CT modelling using a volumetric tracking process. Static placement errors were greatest in a superior-inferior direction (29.5 mm), and the L1–L5 RMS STA error in the sagittal plane ranged from 1.7° to 23.6°. This was larger in flexion-extension than in side-bending. Errors were participant-dependent and unrelated to age and BMI.

The third study [65] compared skin-based MoCap with accelerometers attached over L2, L4, and S1, and 2D quantitative fluoroscopy (QF) at L2–3, L3–4, L4–5 and L5–S1 for contemporaneous recording during flexion and extension in 20 controls. The 95% limits of agreement for L2–S1 between technologies for flexion were: QF vs. MoCap −10.1° to +10.1°, QF vs. accelerometer −9.8° to +9.8°, and accelerometer vs. MoCap −1.0° to 1.1°. For extension, they were: QF vs. MoCap −5.7°–+5.5°, QF vs. accelerometer −6.3° to +6.6°, and for accelerometer vs. MoCap −0.9°–+0.9°. Differences from QF tended to be greater in the lower lumbar spine, and in flexion compared to extension. However, differences between participants were highly variable, confirming the results of the other studies.

Despite the difficulty of placing external markers on the skin over adjacent vertebrae, preventing adjacent intervertebral comparisons from being made between technologies, it can probably be concluded that skin-based marker systems do not measure underlying intervertebral motion accurately. Conversion factors that would improve this do not currently seem to be within reach.

### 3.12. A Taxonomy of Study Types

The need for a taxonomy of study types suggested by these topics is firmly planted in the unknowns of spinal biomechanics and sustained by our erstwhile inability to measure intervertebral motion in vivo. A suggested taxonomy is presented in Table 2.

## 4. Discussion

This review presents the results and trajectory of investigation choices by biomechanics researchers over the past quarter century by identifying the main study types that have addressed the measurement and interpretation of intervertebral motion, as defined by the motion paths and between-level interactions over time, as well as by displacement. As shown in Figure 1, intervertebral motion paths cannot always be expected to be parallel. If they were, the proportional motion that each level contributes would be constant. In real life, they vary during bending tasks, and what keeps trunk motion smooth during a bending task is continuous compensatory interactions between the levels. Access to this continuous motion information makes possible the measurement and interpretation of data from a full spatiotemporal suite of kinematic measures, including laxity, dynamic motion share, velocity, and acceleration. So far, the role of these factors in the clinical management of lumbar spine disorders has only begun to be explored, whereas the documented practical applications of systems have been mainly related to the aims of the 49 studies listed above. Clinicians, especially surgeons and physical therapists, now request investigations to guide treatment for suspected mechanical disorders (such as instability, fusion loosening, adjacent segment disorder, or abnormal post-injury movement) and to aid the planning of cognitive therapy, movement training, manual therapies, and occupational interventions.

In this scoping review, we have attempted to map the key areas of current study and create a taxonomy of vivo lumbar spine intervertebral motion measurement and interpretation from studies published over the past 25 years. The results indicate that research outputs in this field have increased, and the Study types suggest the headline topics. For example, the need to better understand Normal lumbar kinematics is seen as paramount and should encourage the standardisation of available technologies, which at present appear to favour imaging. However, although there are state-of-the-art techniques such as Statistical Parametric Mapping (SPM) for exploring between-level motion path relationships using vector field analysis [66], motor control strategies are inherently interconnected over time. Therefore, a point-by-point evaluation may not be sufficient to fully capture the temporal dependencies underlying coordinated movement. Nevertheless, intervertebral force deformation is also accessible via the kinematic measurement of strains, as demonstrated by Byrne et al. [60].

The attention given to Disc degeneration kinematics is unsurprising given the ubiquitousness of this condition, while the appearance of microgravity’s effects on intervertebral kinematics in space has made an unexpected appearance as Aerospace lumbar kinematics. By contrast, interest in direct intervertebral kinematic measurements using implanted markers and internal sensors placed in situ during surgery may be waning, as, despite the opportunity for greater precision in motion measurement, they did not appear after 2013 in our review.

Given the progress made in disc replacement surgery, including around motion preservation systems, their rare appearance in in vivo kinematics research under post-stabilisation dynamics is surprising and is perhaps attributable to the complexity, scarcity, and cost of fluoroscopic systems for measurement [6,67]. These systems were dominant in the articles found in this review, where technology development has been heavily focused on reducing inaccuracies, imprecision, and, to some extent, radiation dosage. Research into soft tissue artefact measurement, however, has been slow to appear, but now seems largely complete, and some of the questions surrounding the assessment of lumbar orthoses appear to have been addressed.

This leaves nonspecific back pain biomarkers to complete the present taxonomy. Given the heterogeneity and complexity of this condition across biopsychosocial domains, mechanical biomarkers may best contribute to back pain assessment and treatment if considered alongside non-biomechanical mechanisms and interventions such as cognitive behavioural therapy [68,69].

*Limitations and Further Work*

Some studies may have been missed or erroneously excluded by our literature search, especially with respect to the recording and analysis of continuous intervertebral motion data. Non-English language studies that might have contributed were nonetheless excluded. A major weakness for interpretation was the number of studies with small samples, and we recommend replication of those that presented promising results for the discovery of nonspecific back pain biomarkers. Recent scoping reviews relating to the kinematics of the cervical spine and other body joints have called for greater standardisation and sophistication of data recording, image tracking, analysis, and interpolation, and we strongly support this call [6,67]. There is also a particular need to embed automated image registration as a way to reduce the laboriousness of analysis.

Radiation dosage is a cause for concern with some studies, and greater efforts to provide 3D MRI models for biplanar fluoroscopy investigations have been recommended where 3D analysis is judged necessary [70]. We also recommend kinematic studies of the performance of total disc replacements and investigations of adjacent segment disorder, utilising a more complete choice of kinematic measures, including the inclusion of sagittal alignment for structural derangements and extending image acquisition to include recumbent passive motion.

## 5. Conclusions

The research literature in respect of lumbar intervertebral motion is substantial, variable in subject matter, and burdened by a legacy of semantic discrepancies. However, there have been important advances around clinically useful measurement and interpretation, as well as the prospect of others to come. The result of this review challenges the traditional standard of care based on static radiographs, but with no simple, inexpensive, or readily available option to replace it. Hopefully, it will help to clarify available options and provide incentives to pursue the promising ones.

## Figures and Tables

**Table 1:** List of study types.

	Study Type	Number of Studies
1	Intervertebral motion level interactions	4
2	Disc degeneration kinematics	5
3	Implanted markers and internal sensors	5
4	Assessment of lumbar orthoses	2
5	Technology development	10
6	Back pain biomarkers	7
7	Post stabilisation dynamics	2
8	Normative lumbar kinematics	5
9	Intervertebral force deformation	4
10	Aerospace lumbar kinematics	2
11	Soft tissue artefact measurement	3

**Table 2:** Taxonomy of study types.

**Normal biomechanical mechanisms**	**Pathological and injury mechanisms**
Normative lumbar kinematics	Disc degeneration kinematics
Intervertebral motion level interactions	Aerospace lumbar kinematics
Intervertebral force deformation	
**Direct kinematic measurement**	**Spinal stabilisation**
Implanted markers and internal sensors	Assessment of lumbar orthoses
	Post stabilisation dynamics
**Dynamic radiography**	**Clinical markers**
Technology development	Nonspecific back pain biomarkers
Soft tissue artefact measurement	

## Data Availability

The original contributions presented in this study are included in the article/supplementary material. Further inquiries can be directed to the corresponding author.

## Supplementary Materials

The following supporting information can be downloaded at: https://www.mdpi.com/article/10.3390/bioengineering13020239/s1, Table S1: Supplementary file for: Advances in the measurement and interpretation of intervertebral motion in the lumbar spine: A scoping review; Video S1: Digital fluoroscopic video of a standing flexion and return motion sequence in an asymptomatic male showing the angular motion paths of intervertebral levels L2–3 to L5–S1 in Figure 1.

## Abbreviations

The following abbreviations are used in this manuscript:

RoM	Range of motion
ICR	Instantaneous centre of rotation
MSI	Motion sharing inequality
MSV	Motion sharing variability
QF	Quantitative fluoroscopy
CT	Computed tomography
STA	Soft Tissue Artefact
NZ	Neutral Zone
TDR	Total disc replacement
SPM	Statistical parametric mapping
LED	Light-emitting diode
ISS	International Space Station
