# Supplementary material for: Advances in the Measurement and Interpretation of Intervertebral Motion in the Lumbar Spine: A Scoping Review
Source: Bioengineering (Basel). 2026 Feb 18;13(2):239. doi: 10.3390/bioengineering13020239 (PMC12937696; doi:10.3390/bioengineering13020239)
Supplement: Supplementary file 1 [file bioengineering-13-00239-s001.zip › Breen, Alan, et al., 2026 - bioeng suppl tables.pdf]

Supplementary file for: Advances in the measurement and interpretation of intervertebral motion in the lumbar spine: A scoping review

Authors: Alan Breen; Alexander Breen; Jonathan Branney; Alister du Rose, Mehdi Nematimoez.

Journal: Bioengineering – Special Issue: Spine Biomechanics – Year:2026

Title: Recent advances in the measurement and interpretation of intervertebral motion in the lumbar spine: A Scoping review

Content: Accepted studies

Table 1 **Intervertebral motion level interactions**

Author (year)	Country	Purpose of study	Technology	Participants	Measurement	Interpretation	Radiation dose	Significance
Harada et al 2000 (1)	Japan	Compare standing intervertebral flexion and extension at L3-4, L4-5 and L5-S1 for onset and velocity in controls	Cineradiography	10 controls (m)	Order of onset and velocity of angular motion is bottom up for extension and top down for flexion	For standing flexion, onset is sequential and top down. Velocity increases from upper levels	"Radiaton dose of this technique equivalent to 4 plain radiographs	First study to report flexion and extension phase lag.
Breen et al 2020 (2)	UK	Compare continuous proportional motion contributions of lumbar intervertebral levels L2-S1 in chronic back pain patients and controls during controlled active standing flexion and return	2-D quantitative fluoroscopy	8 patients (5m, 3f), 8 controls (5m, 3f)	Level-by-level and continuous between-level differences in patients and controls for: IV-RoM, Motion share level variance (MS(L)V), average motion share/level (AvMS), Motion share/level at maximum bend (MS@max) and L2-S1 Motion share inequality (MSI) and variability (MSV).	Both groups: Motion share consistently highest at L2-3 & L3-4 and lowest at L5-S1. Patients vs controls: L4-5 MS(L)V significantly higher & L5-S1 RoM lower in patients.	NS	Ponential kinematic biomarkers for chronic low back pain
Brownhill et al 2020 (3)	UK	Estimate the main modes of variation in continuous lumbar intervertebral kinematics between back pain patients and controls during passive recumbent flexion, extension and right and left side-bending from L2-3 to L4-5	2-D quantitative fluoroscopy	40 pain free controls (m22, f18) and 40 patients (m22, f18)	801 intervertebral angles in each direction subjected to a smoothing spline using generalised cross-validation, resampled to 40 points per direction and analysed using principal component analysis (PCA)	Three principal components were retained for all motion directions and classified using linear discriminant analysis. The first PC represented a variation in RoM and the second and third variation in motion distribution between joints.Hhowever, statistically significant differences between groups were found for coronal plane motions only, with reduced RoM at the most caudal joint.	NS	Results seem to confirm altered motion sharing between intervertebral joints in people with back . pain.
Nematimoez et al 2023 (4)	Iran	Categorize asymptomatic participants based on clustering of spatial and temporal intervertebral rotation variables during standing lumbar flexion	2-D quantitative fluoroscopy	127 pain free controls (68m, 59f)	Continuous standing flexion & return IV angle data for L2-3 to L5-S1, clustered according to IV RoM, velocity peaking time, velocity magnitude at peaking . time at individual levels and velocity peaking time at grouped levels	For vlocities, more than half of participants had a top-down dominant pattern, based on the lag time between intervertebral velocity peaks, and consistent with Harada (2000)	NS	The hypothesised top-down cascade of intervertebral rotation during standing flexion is supported for a subgroup of healthy controls.

Supplementary file for: Advances in the measurement and interpretation of intervertebral motion in the lumbar spine: A scoping review

Authors: Alan Breen; Alexander Breen; Jonathan Branney; Alister du Rose, Mehdi Nematimoez.

Journal: Bioengineering – Special Issue: Spine Biomechanics – Year:2026

Table 2 **Disc degeneration kinematics**

Author (year)	Country	Purpose of study	Technology	Participants	Measurement	Interpretation	Radiation dose	Significance
Takayanagi et al 2001 (5)	Japan	Compare sitting I/V flexion and return in controls and patients with L4 degenerative spondylo for rotation and translation	Cineradiography	41 patients (m10, f31), 20 controls (NS)	Rotation and translation are simultaneous across levels for controls but disordered for L4-5 spondylo patients.	More severe L4-5 spondylos exhibited decreased translation and less correlation with rotation.	Total radiation exposure as for 2 lateral lumbar roentgenograms	First study to report in vivo spondylolisthesis kinematics.
Hasegawa et al 2008 (6)	Japan	Clarify biomechanial properties of degenerative lumbar discs (DDs) using a passive intraoperative load/deformation measurement system	Prone flx/ext spinous distraction apparatus	22 DD pts (m12, f10) 6 asympt (m3, f3)	Stiffness, absorption energy (AE) & neutral zone (NZ) measured comparing DD sympt with and without height loss <>50%	NZ higher in asymptomatic and <50 height loss groups indicating instability	N/A	DDs with preserved disc height have only latent instability compared to >50% thinned ones.
Breen et al 2018 (7)	UK	Compare the inequality and variability of proportional intervertebral motion sharing (MSI & MSV) between chronic, nonspecific back pain patients and controls during passive recumbent and active weightbearing lumbar motion and the effects of disc degeneration	2-D quantitative fluoroscopy	Passive recumbent: 10 patients (m7, f3 10 controls (m8, f2). Active weightbearing: 10 patients (m6, f4) 10 controls (m6, f4)	MSI, MSV in relation to L2-S1 disc degeneration score in all cohorts	MSI higher in patients than controls in passive recumbent motion and correlated with disc degeneration in patients. . MSV correlated with disc degeneration score in standing flexion.	Effective dose 0.562mSv	Suggestive of links between in vivo disc mechanics and back pain generation.
Dombrowski et al 2018 (8)	USA	Compare the ability of static vs continuous flexion-extension radiography to adequately characterize dynamic instability in patients with degenerative sponylolisthesis (DS)	3D quantitative fluoroscopy and CT models	7 patients (m6, f1) 7 controls (m5, f2)	Magnitude and direction of maximal translation, flexion RoM & slip in neutral position	Continuous dynamic imaging of DS reveals aberrant motion not readily seen on flexion-extension radiographs	Flouroscopy 4.7mSv CT 9.2mSv	Challenge to standard of care for DS
Breen et al 2020 (9)	UK	Determine if early-to-moderate disc degeneration (DD) is associated with intervertebral motion in people without back pain	2D quantitative fluoroscopy and recumbent & weight bearing MRI	10 pain free controls with radiographic DD (m2, f8)	All discs graded for DD using weight bearing MRI by a radiologist and MRI grade correlated with weight bearing and recumbent flexion RoM, translation, laxity, motion sharing inequality (MSI) and variability (MSV).	Weak-moderate significant negative correlation between disc height loss and weight bearing flexion RoM (R=-0.356, p=0.025) and nonsignificant correlation between MRI grade and flexion RoM (R=-0.305, P=0.056).	Effective dose 0.399mSv	Limited evidence of a weak association between early DD and intervertebral motion in people without back pain

Supplementary file for: Advances in the measurement and interpretation of intervertebral motion in the lumbar spine: A scoping review

Authors: Alan Breen; Alexander Breen; Jonathan Branney; Alister du Rose, Mehdi Nematimoez.

Journal: Bioengineering – Special Issue: Spine Biomechanics – Year:2026

Table 3 **Implanted markers & internal sensors**

Author (year)	Country	Purpose of study	Technology	Participants	Measurement	Interpretation	Radiation dose	Significance
Dickey et al 2001 (10)	Canada	Investigate relationship between I/V motion, I/V deformation and pain during a trunk motion battery	LEDs & pedicle screws	9 CLBP patents (m4, f5)	Complex analysis of 3D I/V motion and deformation is linked to 3-layer neural network analysis of pain	Both I/V motion & deformation correlated with reported pain or relief with complex interactions	N/A	Study claims relevance only to candidates for internal fixation testing
Lund et al 2002 (11)	Finland	Compare I/V coupling and asymmetry in CNSLBP patients with controls from published literature	Optical markers attached to pedicle screws percutaneously	27 CLBP patients	I/V flexion-extension ratio, lateral bending asymmetry . & axial rotaton/lateral bending ratio	Lateral bending assymmetry & axial rotation/lateral bending ratio differed from literature controls.	N/A	Study claims relevance only to candidates for internal fixation testing
Gercek et al 2008 (12)	Germany	Determine intervertebral and T11-L2 range and coupling in healthy controls in 3 planes simultaneously	Surface tracking of implanted k-wire sensors	21 controls (11m,19f)	Ultrasound tracking of LEDs attached to spinous-implanted k-wires at T11,T12, L1, L2 for repeatability and accuracy	Single level & total mobility of T12-L2 for flx/ext, lateral bend and axial. Within-subj variation 0.6-0.42% of ranges.	N/A	Mobility less than in literature. Method not recommended . for routine use
Rozumalski et al 2008 (13)	USA	Direct 3D measurement of IV ranges L1-S1 in the sagittal, coronal and transverse planes	Surface tracking of implanted k-wire sensors	10 controls (6m,4f)	Kirshner wires inserted L1-S1 & attached to VICON optical markers tracked against segmented CT scan for 3D RoM in gait, flx.ext, LB & translation	Motion was triaxial with ranges comparable to existing radiographic data (no data during gait available)	N/A	First study in which whole lumbar in vivo IV motion has been measured with bone pins.
MacWilliams et al 2013 (14)	USA	Quantity 3D lumbar vertebral motion during gait in 8 healthy volunteers using motion capture of indwelling bone pin movement ot reflective markers	Spinous Kirshner wires L1-S1 clamped to reflectve marker triads and linked	8 controls (5m, 3f)	Continuous 3D L1-S1 IV rotation recorded in 3 planes during walking	Main motion is mid-lumbar and coronal and during stance and swing phases.	N/A	Valid method for measuring IV motion during gait.

Supplementary file for: Advances in the measurement and interpretation of intervertebral motion in the lumbar spine: A scoping review

Authors: Alan Breen; Alexander Breen; Jonathan Branney; Alister du Rose, Mehdi Nematimoez.

Journal: Bioengineering – Special Issue: Spine Biomechanics – Year:2026

Table 4 **Assessment of lumbar otthoses**

Author (year)	Country	Purpose of study	Technology	Participants	Measurement	Interpretation	Radiation dose	Significance
Kooi et al 2004 (15)	USA	Assess the effect of custom fitted thoracolumbosacral orthosis (TLSO) on vertebral body motion	2-D quantitative fluoroscopy	4 controls (2m,2f)	Free bend flx & ext IV L3-4 & L4-5 comparing no brace, TLSO & TLSO + thigh extender (2 settings).	. IV flx/ext range reduced by 2/3 by brace.	Patient dose m24 rem f75 rem (Repeated once)	First in vivo measurement of . lumbar orthosis effect on IV motion range
Utter et al 2010 (16)	USA	Compare the L3-S1 and individual level flx&ext range wearing no orthotic/soft orthotic/semirigid orthotic/semirigid thoracolumbosacral orthotic	2-D quantitative fluoroscopy	10 controls (6m,4f)	Range of sagittal rotation for each brace condition tested in random order	All orthoses reduced L3-4 & L4-5flexion and none reduced L5-S1 in either flexion or extension		Commercially available soft and semirigid orthotics can have significant effects on lumbar IV motion in flexion.

Supplementary file for: Advances in the measurement and interpretation of intervertebral motion in the lumbar spine: A scoping review

Authors: Alan Breen; Alexander Breen; Jonathan Branney; Alister du Rose, Mehdi Nematimoez.

Journal: Bioengineering – Special Issue: Spine Biomechanics – Year:2026

Table 5 **Technology development**

Author (year)	Country	Purpose of study	Technology	Participants	Measurement	Interpretation	Radiation dose	Significance
Teyhen et al 2005 (17)	USA	Develop technique to measure lumbar spine IV motion using digital fluoroscopy and distortion compensated roentgen analysis (DCRA)	2-D quantitative fluoroscopy	9 controls, 11 LBP (m)	Free bend flx & return L3-4 & L4-5 repeated for reliability (ICC) and (SEM) for rotation & translation.	Average ICCs (Intraimage 0.986 ICC, SEM 0.4-0.7 deg/interimage ICC 0.878, SEM 0.7-1.4deg 0.4-0.7mm)	NS	High repeatability & agreement for standing uncontrolled . sagittal lumbar IV motion
Breen et al 2006 (18)	UK	Assess the reliability, accuracy and radiation dosage of lumbar spine IV motion using controlled bending and automated image tracking	2-D quantitative fluoroscopy	30 controls (m)	Controlled coronal and sagittal passive recumbent L2-S1 twice same .day & calibration model over 30 deg	Coefficient of repeatabilty 1.86-2.9 deg. Accuracy 0.32-0.52 deg.	Effective dose 0.55mSv	Good repeatability and accuracy for unloaded lumbar IV angle motion
Bifulco et al 2012 (19)	Italy	Develop contiinuous calculation method for lumbar sagittal intervertebral instantaneous centre of rotation path and compare average with FCR	2-D quantitative fluoroscopy	3 controls (m)	Smoothing splne incorporated into image tracking and analysis and tested with passive recumbent lumbar flexion image sequences.	ICR trajectory for L2-3 plotted with . smoothing parameters comparing quintic spine and cubic spline with small difference	NS	Smoothing spines are effective in reducing noise and facilitating first derivative (velocity) ICR calculation.
Wang (thesis) 2012 (20)	USA	Quantity 3D lumbar vertebral motion during gait in 8 healthy volunteers using motion capture of indwelling bone pin movement ot reflective markers	Spinous Kirshner wires L1-S1 attached to reflectve marker triads and linked using CT	8 controls (m5, f3)	Continuous 3D L1-S1 IV rotation recorded in 3 planes during walking	Main motion is mid-lumbar coronal and during stance and swing phases.	N/A	Valid method for measuring IV motion during gait.
Nagel et al 2014 (21)	USA	Measure posterior and anterior interbody Green strains from L3-S1 in healthy controls	2-D quantitative fluoroscopy	15 controls (m8, f7)	Continuous 2D L3-S1 standiing flexion and extension for anterior and posterior vertebral body marginal Green strains	Anterior and posterior margin strains greatest at L4-5.	NS	Demonstration of time history of L3-S1 continuous intervertebral Green strains throughout a standing flexion-extension cycle
Davis et al 2015 (22)	USA	Evaluate the detection of breached instability thresholds in low back patients and controls comparing automated dynamic and manual static analysis of weightbearing and recumbent flexon-extension	Automated/controlled 2-D quantitative fluoroscopy vs manual analysis of freebending static fluorograb images	509 patients (m285, f224), 166 controls (m94, f72)	Mean range of continuous IV RoM and translation vs difference in IV angles and translation of static pair in detection of instability defined as >25 deg rotation & >5mm translation	More frequent assignment to instability categories in both samples when analysed by automated/controlled bending method where test-retest reliability is also greater.	NS	Automated/controlled bending continuous analysis provides highter reliability and sensitivity to instability thresholds than static radiography.
Breen et al 2016 (23)	UK	Investigate repeatability, accuracy and radiation dose of 2-D quantitative fluorocopy for measuring flexion and extension translation and finite centre of rotation	2-D passive recumbent quantitative fluoroscopy	20 controls (m9, f11)	Passive recumbent sagittal controlled flexion and extension analysed twice for translation for intraobserver and once by two observers for interobserver agreement. Accuracy by calib model.	Repeatability of translation (ICC) in vivo 0.533-0.935 and 0.421-0.953 for FCR location and was sensitive to radiographic positioning. Accuracy (FCR) 0.1-2.2mm	Effective dose 0.24mSv.	Moderate to high levels of repeatability & accuracy for passive recumbent translation & FCR location
Cheng et al 2016 (24)	USA	Compare the measurement variability of lumbar spine motion when diagnsed using measurements of intervertebral motion taken from standard bending flexion/extension radiographs between uncontrolled and controlled motion	Automated/controlled 2-D lying and standing quantitative fluoroscopy analysis vs free bending standing for L1-S1 flexion extension and side bending	57 patients, 52 controls (gender not reported)	Passive recumbent sagittal(f/e) and coronal controlled and uncontrolled fluoroscopy sequences analysed twice by one observer and 5 times by both for flexion and extension translation for population variability	Measurement variability 26-46% less in controlled bending studies across pooled patient and control populations	NS	Controlled bending fluoroscopic sequences result in less population variability of IV rotation and translation
Andreozzi et al 2020 (25)	Italy	Compare the effects of four different derivative operators (filters) on the preprocessing quality of lumbar vertebral body tracking as reflected in the discrepancies in ICR trajectories in fluoroscopic sequences	2-D quantitative fluoroscopy	1 control (m)	Variability of repeated analysis of Eucliean distances in the passive recumbent ICR trajectories of and L2-3 segment during left and right bending using 4 different pre-processing operators	Best results using Prewitt and Sobel operators. Cerciello and Roberts operators gave the most diffuse results		Indication of optimal image pre-processing operators for lumbar spine fluoroscopic vertebral images
To et sl 2020 (26)	Canada	Determine the intra- and inter-investigator analytic repeatablity of quantitative fluoroscopic measurement of two intervertebral motion biomarkers; Motion sharing inequality and variability (MSI and MSV)	2-D quantitative fluoroscopy	30 pain free controls (15m, 15f)	MSI and MSV analysed from passive recumbent flexion motion from L2-S1 by two independent investigators for intra and inter-investigator ICCs, SEMs and mnimal differences (MDs) for MSI and MSV	Intra-investigator repeatability was substantial for MSI (ICC 0.90) and MSV (ICC 0.78) and inter-investigator (0.93 for MSI and 0.55 MSV).		Confirmation of results in . patients with CNSLBP is now required

Table 6 **Back pain biomarkers**

Author (year)	Country	Purpose of study	Technology	Participants	Measurement	Interpretation	Radiation dose	Significance
Teyhen et al 2007 (27)	USA	Develop I/V kinematic model that characterizes frequently observed movement patterns in patients with LBP	2-D quantitative fluoroscopy	11 LBP (m10, f4) 14 controls (m7, f4)	Free bend flx & return, L3-S1 IV rotation, translation and attainment rate models as LBP predictors.	Model representing midrange disruption in attainment rate accurately discriminated 96% of participants.	NS	Aberrant midrange motion more useful than IV RoM for discriminating individuals with and without LBP.
Cheng et al 2013 (28)	USA	Determine the in vivo biomechanical differences between patients with and without acute low back pain	2-D quantitative fluoroscopy with CT models	10 pain free controls (m5, f5), 10 acute LBP (m5, f5), 10acute LBP with disc degen & spondylosis	Compare average out of plane rotation between groups during flexion-extension incorporating CT generated bone models to compensate for out-of-plane motion	Patients with acute back pain reported had greater out-ot-plane IV motion than healthy . controls	NS	Conventional flexion-extension rediographs inadequate for evaluating motion patterns of lumbar strain
Mellor (thesis) 2014 (29)	UK	Compare continuous passive recumbent flexion-extension and lateral bending IV motion in chronic LBP patients and controls in terms of pain and disability and investigate radiation dose and reliability	2-D quantitative fluoroscopy	40 patients (22m,18f), 40 controls (22m,18f), matched for age/gender/BMI	Continuous 2D L2-L5 recumbent maximum IV rotation & translation range, repeatability & effective radiation effective dose, pain, disability	Weak relationships between kinematics and pain and disability/high reliability/radiation dose comparable with plain radiographs	Effective dose 0.561mSv	Rotation and translation range not clear biomarkers for LBP High reliability and acceptable radiation dosage
Breen et al 2018 (30)	UK	Compare the inequality and variability of proportionl intervertebral motion sharing (MSI and MSV) plus translation in passive recumbent flexion in patients with treatment-resistant back pain and matched controls	2-D quantitative fluoroscopy	37 patients (m23,f14) and 37 controls matched for age and sex	MSI, MSV, laxity and translation L2-S1	MSI significantly higher in patients than controls	NS	Consistent with Breen 2018 and 2020 and supporting recumbent flexion MSI as a CLBP biomarker
Breen et al 2024 (31)	UK	Compare patients with chronic, nonspecific low back pain with a matched group of pain-free controls ford intervertebral restraint as evidenced during passive recumbent flexion and return	2-D quantitative fluoroscopy	17 controls (m10, f7), 17 chronic low back pain patients (m10, f7)	Continuous IV angles for L2-3 to L5-S1 compared according to peaking times of the velocities and magnitudes of individual level angular motion and RoMs	At L5-S1, peak velocity was at 10.82% of the motion path compared with 25.06% in the controls (p = 0.0002), indicating reduced relative restraint at L5-S1 in chronic back pain patients	NS	Findings should be treated with caution pending replication. Future studies should explore relationships with altered disc pressures and biochemistry.
Anderst et al 2025 (32)	USA	Provide a quantitative description of intervertebral motion in the lumbar spine during flexion/extension (F/E) and lateral bending (LB) in individuals with chronc low back. pain (CLBP)	3D quantitative fluoroscopy and CT modelling	125 patients with chronic back pain (m53 f72)	Intervertebral standing flexion-extension and lateral bending ranges from L1-S1 reflect proportional, time-related and age-related contributions, coupling and slip per degree of flexion for some ranges.	Dataset reflects heterogeneity of intervertebral kinematics in individuals with chronic low back pain.	Biplane fluoro 8.8mSv	Further research is needed to identify mechanistic links between kinematics and other biological, behavioral, and clinical features in individuals with CLBP
Xi et al 2025 (33)	China	Quantify and compare the kinematic changes between healthy individuals and patients with recurrent low back pain (LBP) in both rested and fatigued states induced by repetitive lifting	3D quantitative fluoroscopy and CT modelling	23 patients (m11, f12), 19 controls (m11, f12)	Repetitive lifting to fatigue followed by imaging for measurement of time- normalised continuous movement (rot and trans) between the endplate centres of each vertebra and the pelvis (not adjacent segment intervertebral motion)	Significant differences in translation and z-axis rotation occurred in both groups with and without fatigue, but occurred slightly later in the forward-backward bending cycle of L1-L4 to pelvis in patients after . fatigue	NS	May indicate protective compensation or a role in LBP dysfunction.

Supplementary file for: Advances in the measurement and interpretation of intervertebral motion in the lumbar spine: A scoping review

Authors: Alan Breen; Alexander Breen; Jonathan Branney; Alister du Rose, Mehdi Nematimoez.

Journal: Bioengineering – Special Issue: Spine Biomechanics – Year:2026

Table 7 **Post stabilization dynamics**

Author (year)	Country	Purpose of study	Technology	Participants	Measurement	Interpretation	Radiation dose	Significance
Anderst et al 2008 (34)	USA	Preliminary data describing biplanar fluoroscopy tracking of implanted metal markers for measuring post-fusion dynamics	3-D quantitative fluoroscopy	5 post-fusion (m3,f2)	3-D relative rotation of fused and adjacent vertebrae 2,3 & 6m post-fusion & comparison of start/end with continuous recording.	Average precision of implanted bead tracking +/-0.18mm but could not confirm elimination of motion.	NS	Maximum IV displacement may not correspond wth start/end trunk position.
Barrett et al 2015 (35)	Australia	Determine the in vivo kinematics of lumbar functional spinal units after flexible total disc replacements ("In Motion" TDRs) during gait	2-D quantitative fluoroscopy	24 patients (m13, f11)	Mean range of continuous RoM of 10 cycles of motion between upper and lower TDR endplates in coronal and sagittal planes during treadmill walking 6w-5yrs after implantation	DynamicRoM of TDRs at lower end of static range in both planes and at the low end of motion reported for young, healthy individuals	NS	The partial preservation of motion in these lumbar TDRs will help inform standards for conducting wear tests.

Supplementary file for: Advances in the measurement and interpretation of intervertebral motion in the lumbar spine: A scoping review

Authors: Alan Breen; Alexander Breen; Jonathan Branney; Alister du Rose, Mehdi Nematimoez.

Journal: Bioengineering – Special Issue: Spine Biomechanics – Year:2026

Table 8 **Normative lumbar kinematics**

Author (year)	Country	Purpose of study	Technology	Participants	Measurement	Interpretation	Radiation dose	Significance
Mellor et al 2009 (36)	UK	Passive, recumbent, coronal plane L2-S1 quantitative fluoroscopic study of normative intervertebral regularity, symmetery and laxity	2-D quantitative fluoroscopy	30 controls (m)	Range, regularity, symmetry &laxity reported for L2-5 and sEMG investigated in a matched control group who were not imaged	Regularity expressed as correlation of IV with global motion and symmetry as RMS difference between L&R IV RoM - laxity was slope of 1st 10 deg of rotation.	Eff.dose 1.9mSv	First study to demonstrate eMG silence during unloaded intervertebral (L2-S1) motion and passive restraint patterns in coronal plane
du Rose et al 2016 (37)	UK	Explore the relationships between the lumbar inter-vertebral range of motion and paraspinal muscle activity during weight bearing flexion	2-D quantitative fluoroscopy and surface EMG	20 controls (m)	Controlled standing flexion and return fluoroscopy for max IV RoM for correlation with contemporaneous sEMG amplitudes and ratios over longisimus thoracis(TES) , lumborum (LES) and multifidus (LMU)	High sEMG amplitude repeatability and moderate correlations with L4-5 and L5-S1 max RoM	Eff. dose 0.14mSv	First report of relationships between lumbar intervertebral kinematics and spinal muscle electrical activity
Breen et al 2019a (38)	UK	Determine the minimal detectable change in the radiographic measurement of lumbar intervertebral flexion translation comparing 5 different bending protocols	2D quantitative fluoroscopy	55 controls (m34, f21)	Five different standing and recumbent flexion-translation radiographic measurement protocols L2-S1 (pooled data) - static and dynamic - repeated 6 weeks apart - measurement error (MDC^{95})	Measurement error for flexion (MDC^{95}), was greatest (3.36mm) for uncontrolled standing static radiographs	Eff. dose 0.3mSv	Validity of clinical decisions based on sagittal translations depends on the measurement technique error being below the test cutoff
Breen et al 2019b (39)	UK	Determine the intrasubject repeatability of 6 intervertebral motion measures using quantitative fluoroscopy	2D quantitative fluoroscopy	109 controls (m66, f43)	Intrasubject ICC and MDC^{95} and median effective dose for IV best-RoM, laxity, MSI, MSV, flexion translation and anterior disc height change in flexion - standing and lying - coronal and sagittal	Best ICC and MDC^{95} was for disc height change (0.89, 43%) and worst for MSV (0.04, 408%).	Median effective radiation dose per investigation: coronal 0.97 mSv & sagittal 0.66 mSv	Not all lumbar spine quantitative fluoroscopy measures are suitable for longitudinal studies.
Breen et al 2021 (40)	UK	Provide a normative reference database of standardised continuous intervertebral weightbearing and recumbent flexion, extension, left and right bending motion both lying and standing with anonymised patient age, gender and BMI. Geometric data are supplied as vertebral body midplane angles in sequence. (Nested patient/control subgroup study data also supplied)	2D quantitative fluoroscopy	127 pain free controls (m65 , f62): subgroup comparison study 8 back pain patients (m5, f3) and 8 controls (m5, f3)	Level by level continuous proportional angular flexion & return motion and effective dose. Raw vertebral angle data accessuble via the Open Science Framework database https://osf.io/a27py/	Main study: Significant differences between motion paths levels representing normative phenotype. Subgroup study: Significantly greater motion share at L2-L3 in patients during return phase ($p < 0.001$) and less at return phase of L5-S1 ($p<0.012$)	Main study: Mean effective dose: 0.27 mSv (1/4)	In asymptomatic people, consistent intervertebral motion . patterns are evidenced where each level follows its own specific path that changes during the motion.

Supplementary file for: Advances in the measurement and interpretation of intervertebral motion in the lumbar spine: A scoping review

Authors: Alan Breen; Alexander Breen; Jonathan Branney; Alister du Rose, Mehdi Nematimoez.

Journal: Bioengineering – Special Issue: Spine Biomechanics – Year:2026

Table 9 **Intervertebral force-deformation**

Author (year)	Country	Purpose of study	Technology	Participants	Measurement	Interpretation	Radiation dose	Significance
Aiyangar et al 2014 (41)	Switzerland	Demonstrate a dynamic stereo-radiography/CT approach to measuring lumbar spine kinematics including an investigation of use during a functional load-lifting task	High speed orthogonal stereo-radiography L2-S1 linked to CT-generated bone models	8 controls (m)	Continuous 3D L2-S1 IV rotation & translation during during flexed-neutral lifting	Continuous 3D intervertebral motion patterns between the second lumbar vertebra (L2) to the sacrum (S1) were derived characterizing specific level intervertebral kinematics during a loading task	Fluoroscopy 0.6mSv CT 12.33 mSv	Midrange rotational and translational lumbar IV motion during lifting was linear except at L5-S1 and out-of plane motion was negligible
Aiyangar et al 2017 (42)	Switzerland	Map instantaneous centres of rotation during a functional lifting task using a range of weights	3D quantitative fluoroscopy and CT models	14 controls (8m, 6f)	Standing flexion to neutral motion while lifting 10, 20 and 30 lb weights during coronal and saggital L2-S1 fluoroscopic imaging (DSX). Segmentation from CT scans used for bone model frame by frame tracking and ICR mean positions & average ICR migration ranges and directions recorded for each loading condition.Song et al 2021	Average ICR location higher at L2-3 and more dispersed. ICR migration range affected by load magnitude.	Fluoroscopy <3.6mSv CT <12.33mSv	Segment-specific ICR data provide improved input parameters for biomechanical models.
Byrne et al 2019 (43)	USA	Investigate dynamic, level-by-level L2-S1 disc loading and deformation relationships during a common lifting task	3-D quantitative fluoroscopy and CT models	14 controls (m8, f6)	Mapping of dynamic normalised disc height and shear strain from L2-S1 during a lifting task	Level-by-level disc height ith strain patterns and distributions during and at the ends of motion during lifting in 5 disc regions may infer the nature of nucleus pulposus motion	NS	Insight into the segmental restraint mechanisms that accompany dynamic intervertebral loading
Song et al 2021 (44)	China	Explore kinematic changes and symmetry in the laft and right lumbar facet joints under different 3D functional loads during flexion and extension	3D quantitative fluoroscopy and CT models	10 controls (m4 , f6)	Facet joint rotation and translation under 0, 5 &10Kg loads - measuring differences in L3 & L4 rotation angles, mediolateral axis, craniocaudal translations, symmetry of translations and differences in A-P and craniocaudal translations under different weights.	Facet joint kinematics did not change significantly with increased loads except for greater translations at L3 than L4 thought to be due to facet orientation and . symmetry	NS	.."may help to explain the pathogenesis of lumbar spine disease, providing theoretical information to guide clinical surgical procedures"...

Supplementary file for: Advances in the measurement and interpretation of intervertebral motion in the lumbar spine: A scoping review

Authors: Alan Breen; Alexander Breen; Jonathan Branney; Alister du Rose, Mehdi Nematimoez.

Journal: Bioengineering – Special Issue: Spine Biomechanics – Year:2026

Table 10 **Soft tissue artifact measurement**

Author (year)	Country	Purpose of study	Technology	Participants	Measurement	Interpretation	Radiation dose	Significance
Xi et al 2022 (45)	China	1) Quantify the in vivo soft tissue artifacts (STAs) of the human lower back in three-dimensional directions during weight-bearing forward–backward bending and 2) determine the effects of the STAs on the calculated flexion angles between the upper and lower lumbar spines and adjacent vertebrae by comparing the metallic skin markers (SM)- and virtual bone marker (VM)-based measurements using dual fluoroscopy linked to. CT models	3D quantitative fluoroscopy, CT model and reflective markers with lead cores over the L1,3 and 5 spinouses and to the right and left of all vertebrae from L1-S1	6 controls (f)	The A/P, med/lat and prox/distal STAs on lumbar kinematics were quantified throughout the motion using the differences between the skin marker-determined and vertebral measurement-determined kinematics.	Extent of STAs in the lumbar spine differed by anatomical direction, marker location, lumbar segment and bending phases. STAs for flexion ranged from 4.0mm for L1-3 to 13.5mm for L4-5. STAs for extension were less at lower levels	Fluoroscopy 1.2mSv (CT not stated)	The present results for STAs, based on dual fluoroscopic measurements in healthy adult subjects, presented an anatomical direction, marker location, and anatomic segment dependency,
Johnson et al 2024 (46)	USA	Evaluate static marker placement errors, lumbar spine STA from MoCap marker clusters (MMC) and linear relationships between STA and patient characteristics	3D quantitative fluoroscopy, CT and optical motion capture	39 patients with chronic low back pain (m21, f18)	Optical surface marker clusters attached over palpated bone landmarks L1 and 5 and tracked in flx/ext & lat bending using continuous biplane fluoroscopy. Estimation of measurement errors, STA of markers and relationships between STA and patient factors.	Static placement errors were greatest sup/inf (29.5mm). Overall STA RMS error range in sagittal plane in-plane range was $1.7^{o} – 23.6^{o}$). STA tracking errors were greater at L5 than L1 and sagittal errors increased during flexion. Errors were participant-dependent and not related to age & BMI.	Fluoroscopy 8.8mSV CT (average) 9.3mSV	"Given the inaccuracy in marker placement and wide range of patterns of STA, caution is urged when making clinical decisions or when using computational models to estimate spine tissue loading based upon lumbar spine kinematics obtained from skin-mounted markers."
Frey et al 2025 (47)	Canada	Investigate agreement between skin-based accelerometers and optical motion sensors, and quantirative fluoroscopy (QF) measurements of intervertebral flexion-extension motion during standing	2-D quantitative fluoroscopy, skin-based accelerometers and optical motion capture	20 controls (f)	Intervertebral standing: 95% limits of agreement between controlled flexion-extension motion from L2-S1 by QF and contemporaneous surface measurement over L2-3, L4-S1 & L2-S1 by accelerometers and optical motion capture sensors (MoCap). Variation in differences between systems greater towards full bend.	LoAs L2-S1 in degrees: (QF vs MoCap Flx -10.1 to +10.1, Ext -5.7 to +5.5) (QF vs accelerometer Flx -11 0 to +11.9, Ext -6.3 to+ 6.6) (accelerometer vs MoCap Flx -1.0 to +1.1, Ext -0.0 to +0.9). Differences were variable among participants and greatest at end-range.	NS	Skin-based marker systems agreed with each other but can only be used to estimate lumbar spine kinematics and not to measure the underlying intervertebral motion.

Supplementary file for: Advances in the measurement and interpretation of intervertebral motion in the lumbar spine: A scoping review
Authors: Alan Breen; Alexander Breen; Jonathan Branney; Alister du Rose, Mehdi Nematimoez.
Journal: Bioengineering – Special Issue: Spine Biomechanics – Year:2026

Table 11 **Aerospace lumbar kinematics**

Author (year)	Country	Purpose of study	Technology	Participants	Measurement	Interpretation	Radiation dose	Significance
Bailey et al 2022 (48)	USA	Examine the relationship between prolonged exposure to microgravity and the elevated incidence of post spaceflight disc herniation.	2-D quantitative fluoroscopy and MRI	12 ISS crew members (m10, f2)	Factors compared between astronaut incidence of postflight disc hernias: (MRI -1 yr prelaunch, 1w post-flight, 1-2m post-return) Disc water content, DD grade, endplate irregularities, facet atrophy, High intensity zones, herniation, multifidus total and lean X-sectional area and compostion. (QF - preflight and postflight) Maximum flex-ext and lateral bending RoM and translation. (Clinical recording) Outcomes and clinical notes re MRI disc hernia symptoms	Six astronauts developed post flight hernia symptoms/reduced muscle quality with reduced flx-ext RoM at L3-4 & L4-5 and pre-existing end plate irregularities.	NS	Post-spaceflight disc herniation associated with compromised multifidus quality, pre-existing endplate irregulaties and IV kinematic changes that may associate with hernia risk
Breen et al 2023 (49)	UK	Study of the effects of an axial compression skinsuit as a potential lumbar spine microgravity countermeasure in non-terrestrial environments	2-D quantitative fluoroscopy and MRI	20 controls (m)	Measurement of changes in dynamic L2-S1 IV-RoM, translation, disc height, area and volume and lumbar spine length and lordosis during weightbearing and recumbent flexion and extension with and without 4-hour overnight skinsuit use	Participants exhibited significantly more flexion RoM and translation and reduced dynamic disc height at some levels after skinsuit use	Eff. Dose 0.7 mSV for each of 2 studies	Skinsuits might be considered as a microgravity disc hernia countermeasure

References

[1] Harada M, Abumi K, Ito M and Kaneda K 2000 Cineradiographic Motion Analysis of Normal Lumbar Spine During Forward and Backward Flexion *Spine* **25** 1932–7 doi:10.1097/00007632-200008010-00011.

[2] Breen A and Breen A 2020 Dynamic interactions between lumbar intervertebral motion segments during forward bending and return *J. Biomech.* **102** 109603, doi:10.1016/j.jbiomech.2020.109603.

[3] Brownhill K, Mellor F, Breen A and Breen A 2020 Passive intervertebral motion characteristics in chronic mid to low back pain: A multivariate analysis *Méd. Eng. Phys.* **84** 115–25, doi:10.1016/j.medengphy.2020.08.006.

[4] Nematimoez M, Breen A and Breen A 2023 Spatio-temporal clustering of lumbar intervertebral flexion interactions in 127 asymptomatic individuals *J. Biomech.* **154** 111634, doi:10.1016/j.jbiomech.2023.111634.

[5] Takayanagi K, Takahashi K, Yamagata M, Moriya H, Kitahara H and Tamaki T 2001 Using Cineradiography for Continuous Dynamic-Motion Analysis of the Lumbar Spine *Spine* **26** 1858–65, doi:10.1097/00007632-200109010-00008.

[6] Hasegawa K, Kitahara K, Hara T, Takano K, Shimoda H and Homma T 2008 Evaluation of lumbar segmental instability in degenerative diseases by using a new intraoperative measurement system *J. Neurosurg.: Spine* **8** 255–62, doi:10.3171/spi/2008/8/3/255.

[7] Breen A and Breen A 2018 Uneven intervertebral motion sharing is related to disc degeneration and is greater in patients with chronic, non-specific low back pain: an in vivo, cross-sectional cohort comparison of intervertebral dynamics using quantitative fluoroscopy *Eur Spine J* **27** 145–53, doi:10.1007/s00586-017-5155-y.

[8] Dombrowski M E, Rynearson B, LeVasseur C, Adgate Z, Donaldson W F, Lee J Y, A. A and Anderst W J 2018 ISSLS Prize in Bioengineering Science 2018: dynamic imaging of degenerative spondylolisthesis reveals mid-range dynamic lumbar instability not evident on static clinical radiographs *European Spine Journal* **27** 752–62, doi:10.1007/s00586-018-5489-0.

[9] Breen A, Mellor F, Morris A and Breen A 2020 An in vivo study exploring correlations between early-to-moderate disc degeneration and flexion mobility in the lumbar spine *Eur. Spine J.* **29** 2619–27, doi:10.1007/s00586-020-06526-0.

[10] Dickey J P, Pierrynowski M R, Bednar D A and Yang S X 2002 Relationship between pain and vertebral motion in chronic low-back pain subjects *Clin. Biomech.* **17** 345–52, doi:10.1016/s0268-0033(02)00032-3.

[11] Lund T, Nydegger T, Oxland T and Schlenzka, D 2002 Three-Dimensional Motion Patterns During Active Bending in Patients with Chronic Low Back Pain *Spine* **27** 1865–74.

[12] Gercek E, Hartmann F, Kuhn S, Degreif J, Rommens P M and Rudig L 2008 Dynamic Angular Three-Dimensional Measurement of Multisegmental Thoracolumbar Motion In Vivo *Spine* **33** 2326–33, doi:10.1097/brs.0b013e31818096ea.

[13] Rozumalski A, Schwartz M H, Wervey R, Swanson A, Dykes D C and Novacheck T 2008 The in vivo three-dimensional motion of the human lumbar spine during gait *Gait Posture* **28** 378–84, doi:10.1016/j.gaitpost.2008.05.005.

[14] MacWilliams B A, Rozumalski A, Swanson A N, Wervey R A, Dykes D C, Novacheck T F and Schwartz M H 2013 Assessment of Three-Dimensional Lumbar Spine Vertebral Motion During Gait with Use of Indwelling Bone Pins *J. Bone Jt. Surg.* **95** e184, doi:10.2106/jbjs.l.01469.

[15] Kooi D V, Abad G, Basford J R, Maus T P, Yaszemski M J and Kaufman K R 2004 Lumbar Spine Stabilization With a Thoracolumbosacral Orthosis *Spine* **29** 100–4, doi:10.1097/01.brs.0000103945.75275.56.

[16] Basford U A , Anderson, M L , Cunniff, J G , Kaufman, K R , Jelsing, E J , Patrick, T A , Magnuson, D J , Maus, T P , Yaszemski, M J , Anderson M, Cunniff J, Kaufman K, Jelsing E, Patrick T, Magnuson D, Maus T, Yaszemski M and Basford J 2010 Video Fluoroscopic analysis of the effects of three commonly-prescribed off-the-shelf orthoses on vertebral motion. *Spine* **15** E525–9

[17] Teyhen D S, Flynn T W, Bovik A C and Abraham L D 2005 A New Technique for Digital Fluoroscopic Video Assessment of Sagittal Plane Lumbar Spine Motion *Spine* **30** E406–13, doi:10.1097/01.brs.0000170589.47555.c6.

[18] Breen A C, Muggleton J M and Mellor F E 2006 An objective spinal motion imaging assessment (OSMIA): reliability, accuracy and exposure data *BMC Musculoskelet. Disord.* **2006**, **7** 1,

[19] Bifulco P, Cesarelli M, Cerciello T and Romano M 2012 A continuous description of intervertebral motion by means of spline interpolation of kinematic data extracted by videofluoroscopy *J. Biomech.* **45** 634–41, doi:10.1186/1471-2474-7-1.

[20] Wang and S 2012 *In vivo lumbar spine biomechanics: vertebral kinematics, intervertebral disc deformation, and disc loads.* Thesis: Department of Mechanical Engineering, Massachusetts Institute of Technology, Cambridge, Massachusetts.

[21] Nagel T M, Zitnay J L, Barocas V H and Nuckley D J 2014 Quantification of continuous in vivo flexion–extension kinematics and intervertebral strains *Eur. Spine J.* **23** 754–61, doi:10.1007/s00586-014-3195-0.

[22] Davis R J, Lee D C, Wade C and Cheng B 2015 Measurement Performance of a Computer Assisted Vertebral Motion Analysis System *Int. J. Spine Surg.* **9** 36, doi:10.14444/2036.

[23] Breen A and Breen A 2016 Accuracy and repeatability of quantitative fluoroscopy for the measurement of sagittal plane translation and finite centre of rotation in the lumbar spine *Méd. Eng. Phys.* **38** 607–14, doi:10.1016/j.medengphy.2016.03.009.

[24] B. Cheng, A.E. Castellvi, R.J. Davis, D.C. Lee, M.P. Lorio, R.E. Prosko and Wade C 2016 Variability in Flexion Extension Radiographs of the Lumbar Spine: A Comparison of Uncontrolled and Controlled Bending *International Journal of Spine Surgery* 10, doi:10.14444/3020.

[25] Andreozzi E, Pirozzi M A, Fratini A, Cesarelli G and Bifulco P 2020 Quantitative performance comparison of derivative operators for intervertebral kinematics analysis *2020 IEEE Int. Symp. Méd. Meas. Appl. (MeMeA)* **00** 1–6, doi:10.1109/memea49120.2020.9137322.

[26] To D, Breen A, Breen A, Mior S and Howarth S 2020 Investigator analytic repeatability of two new intervertebral motion biomarkers for chronic, nonspecific low back pain in a cohort of healthy controls *Chiropractic and Manual Therapies* **2020** 28, doi:10.1186/s12998-020-00350-5.

[27] Teyhen D S, Flynn T W, Childs J D, Kuklo T R, Rosner M K, Polly D W and Abraham L D 2007 Fluoroscopic Video to Identify Aberrant Lumbar Motion *Spine* **32** E220–9, doi:10.1097/01.brs.0000259206.38946.cb.

[28] Cheng J S, Carr C B, Wong C, Sharma A, Mahfouz M R and Komistek R D 2013 Altered Spinal Motion in Low Back Pain Associated with Lumbar Strain and Spondylosis *Évid.-Based Spine-Care J.* **04** 006–12, doi:10.1055/s-0033-1341640.

[29] Mellor F E 2014 *An evaluation of passive recumbent quantitative fluoroscopy to measure mid-lumbar intervertebral motion in patients with chronic non-specific low back pain and healthy volunteers* Bournemouth University.

[30] Breen A, Mellor F and Breen A 2018 Aberrant intervertebral motion in patients with treatment-resistant nonspecific low back pain: a retrospective cohort study and control comparison *European Spine Journal* **27** 2831–9, doi:10.1007/s00586-018-5666-1.

[31] Breen A, Nematimoez M, Branney J and Breen A 2024 Passive intervertebral restraint is different in patients with treatment-resistant chronic nonspecific low back pain: a retrospective cohort study and control comparison *Eur. Spine J.* 1–15, doi:10.1007/s00586-024-08249-y.

[32] Anderst W, Kim C J, Bell K M, Gale T, Gray C, Greco C M, LeVasseur C, McKernan G, Megherhi S, Patterson C G, Piva S R, Pellegrini C, Schneider M J, Shoemaker J, Smith P, Vo N V and Sowa G A 2025 Intervertebral Lumbar Spine Kinematics in Chronic Low Back Pain Patients Measured Using Biplane Radiography *JOR Spine* **8** e70069, doi:10.1002/jsp2.70069.

[33] Xi X, Zhang L, Yu H, Qin Y, Jia L, Tsai T-Y, Yu Y and Cheng L 2025 Different Spatial Characteristic Changes in Lumbopelvic Kinematics Before and After Fatigue: Comparison Between People with and Without Low Back Pain *Bioengineering* **12** 214, doi:10.3390/bioengineering12030214.

[34] Anderst W J, Vaidya R and Tashman S 2008 A technique to measure three-dimensional in vivo rotation of fused and adjacent lumbar vertebrae *Spine J.* **8** 991–7, doi:10.1016/j.spinee.2007.07.390.

[35] Barrett R S, Lichtwark G A, Armstrong C, Barber L, Scott-Young M and Hall R M 2015 Fluoroscopic Assessment of Lumbar Total Disc Replacement Kinematics During Walking *Spine* **40** 436–42, doi:10.1097/brs.0000000000000787.

[36] Mellor F E, Muggleton J M, Bagust J, Mason W, Thomas P W and Breen A C 2009 Midlumbar Lateral Flexion Stability Measured in Healthy Volunteers by In Vivo Fluoroscopy *Spine* **34** E811–7, doi:10.1097/brs.0b013e3181b1feba.

[37] A. du Rose and Breen A 2016 Relationships between Paraspinal Muscle Activity and Lumbar Inter-Vertebral Range of Motion *Healthcare* 4, doi:10.3390/healthcare4010004.

[38] Breen A, Claerbout E,. Hemming R. Ayer R, and Breen A 2019 Comparison of intra subject repeatability of quantitative fluoroscopy and static radiography in the measurement of lumbar intervertebral flexion translation *Scientific Reports* **9** 19253, doi:10.1038/s41598-019-55905-1.

[39] Breen A, Hemming R, Mellor F and Breen A 2019 Intrasubject repeatability of in vivo intervertebral motion parameters using quantitative fluoroscopy *Eur Spine J* **28** 450–60, doi:10.1007/s00586-018-5849-9.

[40] Breen A, Carvalho D D, Funabashi M, Kawchuk G, Pagé I, Wong A Y L and Breen A 2021 A Reference Database of Standardised Continuous Lumbar Intervertebral Motion Analysis for Conducting Patient-Specific Comparisons *Front. Bioeng. Biotechnol.* **9** 745837, doi:10.3389/fbioe.2021.745837.

[41] Aiyangar A K, Zheng L, Tashman S, Anderst W J and Zhang X 2014 Capturing Three-Dimensional In Vivo Lumbar Intervertebral Joint Kinematics Using Dynamic Stereo-X-Ray Imaging *J. Biomech. Eng.* **136** 011004, doi:10.1115/1.4025793.

[42] Aiyangar A, Zheng L, Anderst W and Zhang X 2017 Instantaneous centers of rotation for lumbar segmental extension in vivo *J. Biomech.* **52** 113–21, doi:10.1016/j.jbiomech.2016.12.021.

[43] Byrne R M, Aiyangar A K and Zhang X 2019 A Dynamic Radiographic Imaging Study of Lumbar Intervertebral Disc Morphometry and Deformation In Vivo *Sci. Rep.* **9** 15490, doi:10.1038/s41598-019-51871-w.

[44] Song Y, Wen W, Xu J, Zhang Z, Han Y, Li K, Wang X, Xu H, Liu J and Miao J 2021 Kinematic Characteristics and Biomechanical Changes of Lower Lumbar Facet Joints Under Different Loads *Orthop. Surg.* **13** 1047–54, doi:10.1111/os.12894.

[45] Xi X, Ling Z, Wang C, Gu C, Zhan X, Yu H, Lu S, Tsai T-Y, Yu Y and Cheng L 2022 Lumbar segment-dependent soft tissue artifacts of skin markers during in vivo weight-bearing forward–Backward bending *Front. Bioeng. Biotechnol.* **10** 960063, doi:10.3389/fbioe.2022.960063.

[46] Johnson M E, LeVasseur C, Gale T, Megherhi S, Shoemaker J, Pellegrini C, Gray E C, Smith P and Anderst W J 2024 Lumbar spine marker placement errors and soft tissue artifact during dynamic flexion/extension and lateral bending in individuals with chronic low back pain *J. Biomech.* **176** 112356, doi:10.1016/j.jbiomech.2024.112356.

[47] Frey M, Breen A, Rix J, and Carvalho D D. 2025 Concurrent validity of skin-based motion capture systems in measuring dynamic lumbar intervertebral angles *J. Biomech.* **180** 112503, doi:10.1016/j.jbiomech.2025.112503.

[48] Lotz J C, Bailey J F, Nyayapati, P , Johnson, G T A , Dziesinski, L , Scheffler, A W , Crawford, R , Scheuring, R , O'Neill, C W , Douglas Chang, Hargens, A R , 2022 Biomechanical changes in the lumbar spine following spaceflight and factors associated with postspaceflight disc herniation *The Spine Journal* **2** 197–206, doi:10.1016/j.spinee.2021.07.021.

[49] Breen A, Carvil P, Green D, Russomano T and Breen A 2023 Effects of a microgravity SkinSuit on lumbar geometry and kinematics *European Spine Journal* **32** 839–47, doi:10.1007/s00586-022-07454-x.
